# Association between childhood maltreatment and the prevalence and complexity of multimorbidity: A cross-sectional analysis of 157,357 UK Biobank participants

**DOI:** 10.1177/2235042X10944344

**Published:** 2020-07-31

**Authors:** Peter Hanlon, Marianne McCallum, Bhautesh Dinesh Jani, Ross McQueenie, Duncan Lee, Frances S Mair

**Affiliations:** 1 General Practice and Primary Care, Institute of Health and Wellbeing, University of Glasgow, UK; 2 School of Mathematics and Statistics, The mathematics and Statistics Building, University of Glasgow, University Place, Glasgow, UK

**Keywords:** Multimorbidity, child maltreatment, social isolation, frailty

## Abstract

**Background:**

Child maltreatment is associated with long-term conditions (LTCs) in adulthood. Its relationship to multimorbidity (≥2 LTCs) is less clear. We explore the relationship between child maltreatment, multimorbidity and factors complicating management.

**Methods:**

Cross-sectional analysis of 157,357 UK Biobank participants. Experience of four maltreatment types (physical/sexual/emotional/neglect) was identified. We explored the relationship between type, number and frequency of maltreatment and LTC count (0, 1, 2, 3, ≥4) using multinomial logistic regression. Binary logistic regression assessed the relationship between maltreatment and self-rated health, loneliness, social isolation, frailty and widespread pain in those with multimorbidity, adjusting for sociodemographics and lifestyle factors.

**Results:**

52,675 participants (33%) experienced ≥1 type of maltreatment; 983 (0.6%) experienced all four. Type, frequency and number of types of maltreatment were associated with higher LTC count. People experiencing four types of maltreatment were 5 times as likely to have a LTC count of ≥4 as those experiencing none (odds ratio (OR): 5.16; 99% confidence interval (CI): 3.77-7.07). Greater number of types of maltreatment was associated with higher prevalence of combined physical/mental health LTCs (OR: 2.99; 99% CI: 2.54–3.51 for four types of maltreatment). Compared to people who reported no maltreatment, people experiencing all four types of maltreatment were more likely to have poor self-rated health (OR: 3.56; 99% CI: 2.58–4.90), loneliness (OR: 3.16; 99% CI: 2.17–4.60), social isolation (OR: 1.45; 99% CI: 1.03–2.05), widespread pain (OR: 3.19; 99% CI: 1.87–5.44) and frailty (OR: 3.21; 99% CI: 2.04–5.05).

**Conclusion:**

Peoplewith a history of maltreatment have higher LTC counts and potentially more complicated management needs reinforcing calls for early intervention.

## Introduction

Child maltreatment describes ‘*all types of physical and/or emotional ill-treatment, sexual abuse, neglect, negligence and commercial or other exploitation*’^1^ resulting in actual or potential harm. Child maltreatment is frequently categorised as neglect, physical, emotional or sexual abuse. It is associated with poor health outcomes in adulthood.^2,3^ It is a common and under-recognised problem, for example the World Health organisation estimates that one in four adults report having experienced physical abuse as a child.^1^ There has been growing interest not just into child maltreatment but the wider impact of Adverse Childhood Experiences (ACES)^3^ which include child maltreatment as well as domestic violence, parental abandonment, parent with a mental health condition, member of the household in prison, adult in household experiencing drug or alcohol problems. These have strong associations with multiple poor social and health outcomes and result in a significant economic burden.^4^ Experience of maltreatment is associated with increased prevalence of risk factors for chronic disease (e.g. smoking, obesity)^3^ and a range of different physical and mental health conditions.^2,3^ If more than one type of maltreatment is experienced, then the risk of developing a mental or physical health condition is greater.^2,3^

A life course approach to epidemiology recognises that exposure to risk factors in early life can initiate disease processes, although manifestation of disease may not be seen for years.^5^ This approach to understanding the development of chronic disease also recognises that there are particularly sensitive times of development when exposure has a greater impact. This approach helps clarify why adversity in childhood (including maltreatment) can result in increased chronic disease in adulthood. Firstly, the impact of ACES on mental health, and the later adoption of unhealthy behaviours in adulthood, is one possible reason for the increase in chronic disease.^6^ In addition, there is growing evidence that ‘toxic’ stress^7,8^ (including abuse and neglect) causes an increased allostatic load, as well as shortening of chromosome telomere length, which may impair developing neurological, endocrine and immune systems,^2,7,9^ and alter gene expression.^10^ This ‘toxic’ stress also affects cognitive, social and emotional development.^2,7^ The links are seen either with cumulative exposure or with exposure at particularly sensitive times for brain development.^8,9^ While the detailed understanding of biological pathways requires further clarification, there is enough evidence for the impact of childhood adversity on long-term health to justify intervening.^10^

Multimorbidity (the presence of two or more long-term conditions (LTCs))^11^ is a major global challenge.^11^ Multimorbidity is socially patterned, being more prevalent, and more likely to include psychological as well as physical LTCs, in more socioeconomically deprived areas.^12^ This observation is only partially explained by differences in health behaviours,^13^ suggesting additional factors are likely to underlie these inequalities.^14^

Given the association between child maltreatment and multiple individual LTCs, it would be expected that it would be associated with an increased prevalence of multimorbidity.^15–18^ However, studies examining prior traumatic childhood experience, including maltreatment, and adult multimorbidity have varied in how maltreatment was measured.^16,17^ In addition, while multimorbidity is associated with previous maltreatment, it has previously been measured as a binary outcome of two or more conditions,^16–18^ or in the context of combined physical and mental health problems,^19^ or chronic pain.^15^ It is not clear what the association is between child maltreatment and increasing numbers of LTCs (rather than a binary cut-off of >2 LTCs).

While multimorbidity is associated with poor health outcomes such as mortality and hospitalisation,^20,21^ people with multimorbidity are highly heterogenous.^11,22^ How well people manage their multimorbidity, and the impact on their quality of life, is not solely dependent on the number of LTCs experienced but also with other factors that increase complexity.^23^ This study draws on burden of treatment theory^24^ which recognises the complex interplay between biological, social and psychological factors in both the work (or treatment burden) generated by LTCs and patient capacity to carry it out. An imbalance between patient capacity and workload is recognised to lead, over time, to cumulative complexity resulting in poorer access to healthcare, self-management and treatment outcomes.^23,24^

As well as social factors, the types of LTCs impact both treatment burden and capacity. Having a mental health condition as one of the LTCs increases rates of unplanned hospital admissions,^21^ and reduces patient enablement,^25^ resulting in both increased treatment burden and reduced capacity. The relationship between child maltreatment and the extent (in terms of LTC count) and the complexity (presence of factors that may increase treatment burden or reduce capacity) of multimorbidity have not been examined.

We hypothesised that several factors known to impact treatment burden and patient capacity (loneliness, social isolation, mental health as one of the LTCs, widespread pain and frailty)^2,21,23,26–29^ may also be associated with childhood maltreatment. It is recognised that there are differences in the associations between the different types of child maltreatment and emotional and social outcomes in adulthood.^4^ In addition, the associations between adversity in childhood (including maltreatment) and increased prevalence of some individual LTCs, and unhealthy behaviours, are increased if more than one type of adversity is experienced.^3^

Therefore, there may be an association between type, and frequency of child maltreatment and adult multimorbidity. This association may not just be with increasing numbers of LTCs in adulthood but with factors that complicate both the experience of multimorbidity for patients, as well as the experience of practitioners seeking to manage them. This could have potential resource implications if areas with a higher proportion of the population has experienced childhood maltreatment are associated with increased disease burden, and factors that complicated its experience and management.^23,26,24,30–32^

Recognising that multiple factors influence multimorbidity management, experiences and outcomes, we aim to examine the association of experience of childhood maltreatment with

- LTC count reported in adulthood
- the presence of combined mental and physical multimorbidity
- potential complicating factors (widespread pain, loneliness, social isolation and frailty)^26–28,33^
- self-rated health (a measure of patient experience of multimorbidity).

## Methods

### Study design and participants

**Figure 1.**
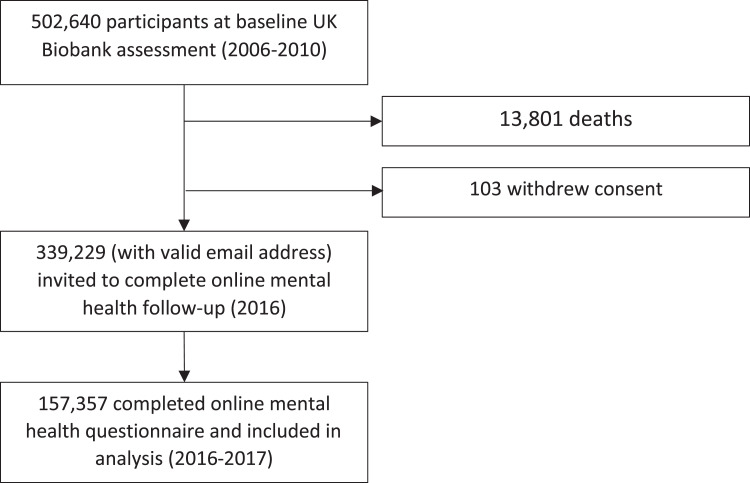
Selection of study participants. *Note:* Flow diagram showing the number of participants recruited at baseline and the number completing the online follow-up. Data on demographics, lifestyle, multimorbidity health status were assessed at the baseline assessment centre. Experience of child maltreatment was ascertained from the online follow-up questionnaire.

This is a cross-sectional analysis of participants in the UK Biobank research cohort. UK Biobank recruited 502,640 adult participants by postal invitation between 2006 and 2010. Eligible participants (registered with a UK general practice and living within 20 miles of an assessment centre) were invited to attend by postal invitation. Response rate was 5%. Each participant attended one of 22 assessment centres across England, Scotland and Wales, completed a touchscreen questionnaire, nurse-led interview and had physical measurements. All participants gave informed consent for data collection and analysis. A subsample of the original cohort (*n*= 157,357) completed a subsequent online mental health follow-up questionnaire between 2016 and 2017 that contained questions pertaining to childhood maltreatment. The analysis presented here is based on this subsample (Figure 1). This study is part of UK Biobank project 14151 (NHS National Research Ethics Service (16/NW/0274)).

### Procedures

The following four questions from the online mental health follow-up questionnaire, taken from the Childhood Trauma Questionaire,^34^ were used to identify participants experiencing maltreatment in childhood: *Physical abuse* (‘When I was growing up…People in my family hit me so hard that it left me with bruises or marks’), *Emotional abuse* (‘When I was growing up…I felt that someone in my family hated me’), *Sexual abuse* (‘When I was growing up…Someone molested me (sexually)’) and *Neglect* (‘When I was growing up…There was someone to take me to the doctor if I needed it’). Possible responses were never true, rarely true, sometimes true, often true and very often true, and prefer not to answer. ‘Prefer not to answer’ was treated as missing for the analyses.

We quantified experience of maltreatment in several ways. Initially, to assess the impact of any experience of maltreatment, we treated each separate type of maltreatment as a binary variable. For physical, emotional and sexual abuse, ‘never’ was taken as the absence of maltreatment and any positive response treated as the presence of maltreatment. For the neglect question, ‘never true’, ‘rarely true’ and ‘sometimes true’ were used to identify potential neglect, and ‘often true’ or ‘very often true’ the absence of neglect. This categorisation was chosen to reflect the higher cut-off for neglect within the Child Trauma Questionnaire.^34^

Next, we considered the frequency of each separate type of maltreatment. We collapsed the possible responses for physical, sexual and emotional abuse into three categories: often (very often, often), occasionally (rarely or sometimes) and never. Neglect was categorised as often (‘never true’ or ‘rarely true’ in response to ‘there was someone to take me to the doctor if I needed it’), occasionally (‘sometimes’) and never (‘often true’ of ‘very often true’).

Finally, we summed the binary variables for each of the four questions to assess a count of different types of maltreatment experienced (0, 1, 2, 3 or 4).

Sociodemographic and lifestyle characteristics were based on baseline assessment centre data. Age was treated as continuous. Socioeconomic deprivation was assessed using Townsend scores: an area specific measure of socioeconomic deprivation based on preceding national census data. Body mass index (BMI) was categorised into underweight (<18.5 kg/m^2^), normal weight (18.5–25 kg/m^2^), overweight (25–30 kg/m^2^) and obese (>30 kg/m^2^). Smoking was categorised as current, ex- and non-smoker. Alcohol intake was based on self-reported frequency of alcohol intake (never/special occasions only; one to three times per month; one to four times per week; daily/almost daily).

### Outcomes

The first outcome considered was LTC count. At baseline, all participants were interviewed by a study nurse in which they were asked to list all of their long-term health conditions. We quantified multimorbidity based on a simple count of these self-reported conditions. The conditions used to assess multimorbidity for this analysis were taken from a list of 43 LTCs originally established for a large epidemiological study in Scotland, through systematic review, the Quality and Outcomes Framework, NHS Scotland and an expert panel^12^ and subsequently amended for UK Biobank.^35^ The number of LTCs was summed and then categorised to give LTC counts of 0, 1, 2, 3 and 4 or more. From this list of 43 LTCs, 7 mental health conditions were identified (depression, anxiety, bipolar affective disorder/schizophrenia, bulimia/anorexia nervosa, alcohol dependence, other psychoactive substance misuse and dementia) to allow assessment of the proportion of multimorbidity with a mental health component.

An additional group of outcomes were selected from data available from baseline assessment: poor self-rated health, loneliness, social isolation, widespread pain and frailty. These variables were selected as markers of increased complexity, particularly in the management of multimorbidity. Each of these factors is likely to be associated with multimorbidity but describes distinct entities from a simple count of LTCs. The relationship between childhood maltreatment and these variables was assessed in participants with two or more LTCs, to assess the association between childhood maltreatment and factors that may complicate the lives and care of those with multimorbidity. Self-rated health was assessed by ‘*In general how would you rate your overall health?*’ and categorised as excellent/good/fair versus poor. Loneliness was assessed by two questions. ‘*Do you often feel lonely?*’ (Yes/no response) and ‘*How often are you able to confide in someone*’ (never/almost never versus almost daily to once every few months). Participants were considered lonely if they answered positively to both questions for consistency with previously published literature on loneliness in UK Biobank.^27,28^ Social isolation was assessed by scales used in previous UK Biobank studies comprising three questions. ‘*Including yourself, how many people are living in your household*’ (deemed positive if living alone). ‘*How often do you visit friends or family or have them visit you*’ (positive if less than once per month). And ‘*Which if the following (leisure/social) activities do you engage in once a week or more often*’ (positive if no leisure activities). Participants were considered socially isolated if they answered positively to two or more out of three questions.^27^ Widespread pain was defined as pain described as ‘*all over the body*’ and being present ‘*for a period of greater than 3 months*’.^36^ Frailty was assessed using an adaptation of the frailty phenotype model for UK Biobank, as described elsewhere.^26^ Briefly, frailty was assessed based on five characteristics – low grip strength, self-reported exhaustion, slow walking speed, weight loss and low physical activity. Participants were deemed frail if they met three or more criteria, pre-frail if they fulfilled one or two criteria and robust if no criteria were met.

These data were taken from the baseline assessment centre data (2006–2010, contemporaneous with the assessment of sociodemographic characteristics and LTC count).

### Statistical analysis

All analyses were planned prior to inspection of the data according to STROBE guidelines (https://www.strobe-statement.org).

First, sociodemographic characteristics were summarised descriptively with counts and percentages, comparing participants experiencing each type of maltreatment to those reporting no experience of maltreatment. Differences in baseline characteristics between participants with and without each type of abuse were tested using *χ* ^2^ test for categorical variables and Mann–Whitney *U* test for continuous variables. These characteristics were also compared between participants completing the online follow-up questionnaire and those who did not.

Next, to descriptively summarise the relationship between maltreatment and number of LTCs, we assessed the number of LTCs in those experiencing childhood maltreatment compared to those who did not. Counts and percentages were used to summarise number of LTCs (0, 1, 2, 3 and 4 or more) in participants with each type of maltreatment as a binary variable, each type as an ordinal variable based on frequency, and as an ordinal variable of number of different types of maltreatment experienced.

We then assessed the relationship between maltreatment and multimorbidity adjusting for demographic and lifestyle factors as potential confounders. We used multinomial logistic regression to model the relationship between number of different types of maltreatment experienced (0, 1, 2, 3 or 4) and LTC count, adjusting for age, sex, socio-economic deprivation, BMI, smoking and alcohol. Multinomial logistic regression allows simultaneous estimation of the probability of different outcomes. Separate odds ratios (ORs) were calculated for 1, 2, 3 and 4 or more LTCs.

To assess the relationship between maltreatment and the presence of a mental health condition in the context of multimorbidity, we used binary logistic regression to model the relationship between number of different types of maltreatment and the presence of a mental health condition. We calculated the OR for the presence of any mental health condition adjusting for age, sex, socio-economic deprivation, BMI, smoking, alcohol and number of physical LTCs.

Our final analysis was to assess the relationship between maltreatment and a range of factors that may complicate patient experience and clinical management in the context of multimorbidity. We restricted theseanalyses to participants with two or more LTCs. We used binary logistic regression models to assess the relationship between childhood maltreatment (type, total number and frequency) and self-rated health, loneliness, social isolation and widespread pain. Multinomial logistic regression was used to model the relationship with frailty and pre-frailty. These models were adjusted for age, sex, socioeconomic deprivation, BMI, smoking and alcohol frequency. To reduce the risk of false positive results on testing multiple outcomes, we calculated 99% confidence intervals (CIs) based on a Bonferroni correction. These models were repeated for each type of maltreatment as a binary variable and for the cumulative count of types of maltreatment. As a sensitivity analysis, we also ran these analysis on the full sample, also adjusting for number of LTCs. All analyses were performed using R (version 3.5.1).

## Results

### Population

Of the 502,640 participants recruited to UK Biobank and completing baseline assessments between 2006 and 2010, 157,357 (31%) completed the mental health follow-up questionnaire in 2016–2017 and were included in the analysis. Participants completing the follow-up questionnaire were less socio-economically deprived and had less multimorbidity compared to the original sample (Online Supplementary Table 1).

### Experience of childhood maltreatment

The responses to the four questions used to identify experience of childhood maltreatment are summarised in Tables 1 and 2. A total of 52,675 (33%) participants answered positively to at least one of the questions, with 34,393 (22%) reporting experiencing one type, 13,219 (8%) reporting two types, 4080 (3%) reporting three types and 983 (0.6%) reporting having experienced all four types of maltreatment.

**Table 1.** Number of participants reporting each type of maltreatment: Presence or absence of each type of maltreatment.

	Physical abuse, *n* (%)	Emotional abuse, *n* (%)	Sexual abuse, *n* (%)	Neglect, *n* (%)
Present	29,799 (18.9)	24,552 (15.6)	13,647 (8.7)	9005 (5.7)
Absent	127,200 (80.8)	132,351 (84.1)	141,858 (90.2)	147,278 (93.6)
Missing (prefer not to answer)	358 (0.2)	454 (0.3)	1852 (1.2)	1074 (0.7)

**Table 2.** Number of participants reporting each type of maltreatment: Reported frequency of each type of maltreatment.

	Physical abuse, *n* (%)	Emotional abuse, *n* (%)	Sexual abuse, *n* (%)	Neglect, *n* (%)
Often	2338 (1.5)	4577 (2.9)	1459 (0.9)	4683 (3.0)
Occasionally	27,461 (17.5)	19,975 (12.7)	12,188 (7.7)	4322 (2.7)
Never	127,200 (80.8)	132,351 (84.1)	141,858 (90.2)	147,278 (93.6)
Missing (prefer not to answer)	358 (0.2)	454 (0.3)	1852 (1.2)	1074 (0.7)

### Baseline characteristics

The baseline sociodemographic characteristics, lifestyle factors and LTC count of participants reporting each type of maltreatment are summarised in Table 3. A higher proportion of participants reporting each type of maltreatment were from more socioeconomically deprived areas, were current smokers, were never or occasional alcohol drinkers and were obese compared to participants who did not report any experience of childhood maltreatment. Emotional maltreatment, sexual maltreatment and neglect were reported by a higher proportion of females than males.

**Table 3.** Baseline demographic and lifestyle factors and reported experience of maltreatment in childhood.^a^

Variable	No childhood maltreatment (*n* = 102,798)	Specific types of childhood maltreatment			
		History of physical abuse (*n* = 29,799)	History of emotional abuse (*n* = 24,552)	History of sexual abuse (*n* = 13,647)	History of neglect (*n* = 9005)
Age, median (IQR)	58 (51–62)	55 (48–61)	54 (48–60)	56 (49–61)	58 (51–63)
Sex					
Female (%)	57,279 (56)	15,394 (52)	15,857 (65)	9697 (71)	5437 (60)
Socioeconomic deprivation (%)					
Quintile 1	24,634 (24)	6072 (20)	4525 (19)	2570 (20)	1718 (19)
Quintile 2	22,960 (22)	5952 (20)	4441 (18)	2518 (19)	1669 (19)
Quintile 3	21,574 (21)	5933 (20)	4793 (20)	2646 (19)	1740 (19)
Quintile 4	19,340 (19)	6128 (21)	5414 (22)	2972 (22)	1880 (21)
Quintile 5	14,168 (14)	5668 (19)	5350 (22)	2920 (21)	1986 (22)
Missing	122	46	29	21	12
Smoking (%)					
Current	6362 (6)	2954 (10)	2624 (11)	1324 (9)	918 (10)
Previous	34,152 (33)	11,874 (40)	9344 (39)	5536 (41)	3401 (38)
Never	62,063 (60)	14,881 (50)	12,206 (50)	6759 (50)	4647 (52)
Missing	221	90	78	28	39
Alcohol (%)					
Never/occasional	13,999 (14)	4818 (16)	4681 (19)	2637 (19)	2095 (23)
1–3/month	10,998 (11)	3485 (12)	2994 (12)	1660 (12)	1051 (12)
1–4/week	53,288 (52)	14,834 (50)	11,560 (47)	6350 (47)	4169 (46)
Daily/almost daily	24,445 (24)	6632 (22)	5290 (22)	2987 (22)	1679 (19)
Missing	68	30	27	13	11
BMI (%)					
<18.5	610 (0.6)	123 (0.4)	150 (0.6)	72 (0.5)	55 (0.6)
18.5–25	39,557 (39)	9447 (32)	8734 (36)	4857 (36)	2795 (31)
25–30	43,224 (42)	12,649 (43)	9784 (40)	5407 (40)	3774 (43)
>30	18,866 (18)	7322 (25)	5655 (23)	3189 (24)	2276 (26)
Missing	541	258	229	122	105
No. of LTCs (%)					
0	41,593 (41)	10,690 (36)	8459 (35)	4767 (35)	2831 (32)
1	34,657 (34)	9899 (33)	8031 (33)	4353 (32)	2913 (33)
2	17,158 (17)	5359 (18)	4588 (19)	2533 (19)	1758 (20)
3	6384 (6)	2356 (8)	2018 (8)	1161 (9)	855 (10)
≥4	2843 (3)	1411 (5)	1365 (6)	794 (6)	610 (7)
Missing	163	84	91	39	38

IQR: interquartile range; BMI: body mass index; LTC: long-term condition.

^a^Difference in baseline characteristics between participants with and without each type of abuse tested using Chi-squared test for categorical variables and Mann-Whitney U test for continuous variables. P<0.001 for all baseline characteristics and child maltreatment.

**Figure 2.**
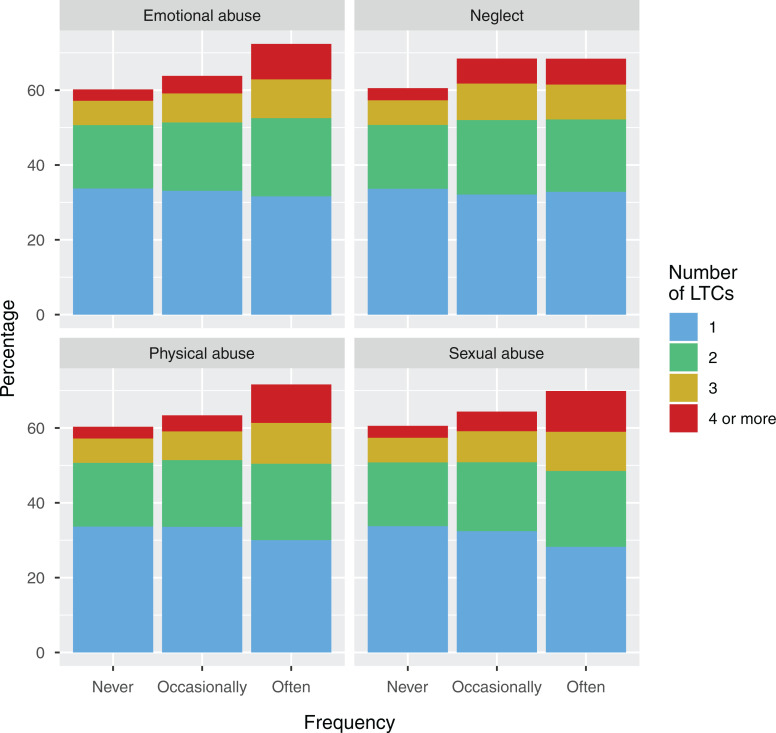
Frequency of maltreatment and multimorbidity. *Note:* For each type of maltreatment, this figure displays the proportion of participants with 1, 2, 3 or 4 or more LTCs by frequency of experience of maltreatment. Increasing frequency of maltreatment is associated with a greater number of LTCs. LTC: long-term condition.

**Figure 3.**
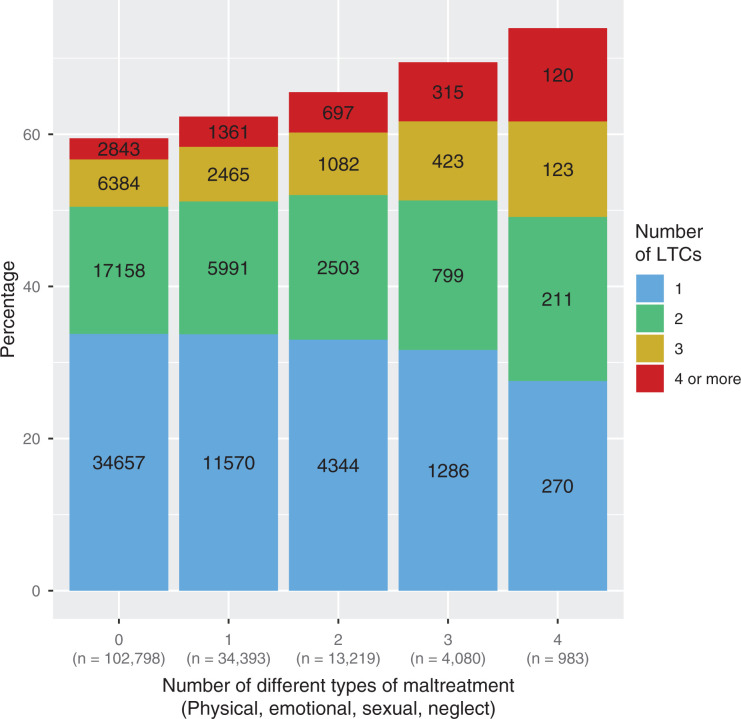
Count of childhood maltreatment and multimorbidity. *Note:* For participants experiencing different total types of maltreatment (0, 1, 2, 3 or 4 types of maltreatment), this figure shows the number of LTCs. Experiencing a greater number of types of maltreatment is associated with a greater number of LTCs. LTC: long-term condition.

Both higher frequency of childhood maltreatment and experience of a greater number of different types of maltreatment were associated with higher LTC count. These relationships are illustrated in Figures 2 and 3, respectively. After adjusting for age, sex, socio-economic deprivation, BMI, smoking and alcohol frequency, greater number of types of maltreatment experienced remained significantly associated with greater LTC count (Table 4). Participants who had experienced all four types of maltreatment were five times as likely to have an LTC count of four or more than people reporting no experience of maltreatment (OR: 5.16; 99% confidence interval (CI): 3.77–7.07).

**Table 4.** Association between number of types of childhood maltreatment and number of LTCs (odds ratios and 99% confidence intervals).^a^

Number of types of maltreatment	Number of LTCs			
	1 LTC	2 LTCs	3 LTCs	4 or more LTCs
0	(ref)	(ref)	(ref)	(ref)
1	1.09 (1.05–1.13)	1.15 (1.10–1.21)	1.26 (1.18–1.35)	1.52 (1.39–1.67)
2	1.18 (1.11–1.25)	1.40 (1.30–1.50)	1.59 (1.44–1.76)	2.22 (1.96–2.50)
3	1.27 (1.14–1.41)	1.60 (1.42–1.81)	2.20 (1.88–2.57)	3.37 (2.81–4.03)
4	1.28 (1.02–1.61)	1.97 (1.53–2.53)	2.80 (2.07–3.78)	5.16 (3.77–7.07)

LTC: long-term condition.

^a^Odds ratios are from a multinomial logistic regression model. This model simultaneously calculates odds ratios for the association between an exposure (number of types of maltreatment) and multiple categories of an outcome (number of LTCs). The model is adjusted for age, sex, socioeconomic deprivation, BMI, smoking and alcohol intake. 99% confidence intervals are presented based on a Bonferroni correction for a 5-level outcome variable.

**Figure 4.**
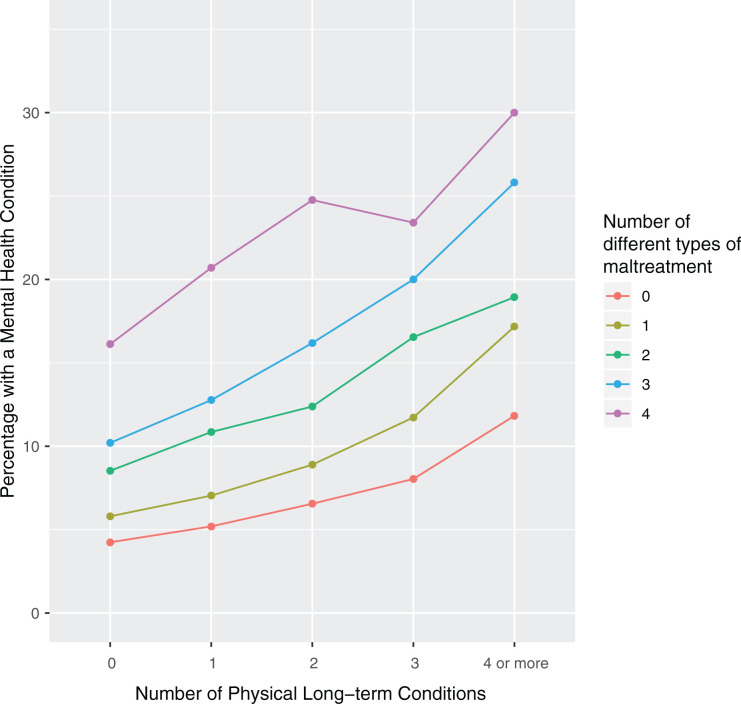
Multimorbidity with a mental health component. *Note:* For participants experiencing different total types of maltreatment (0, 1, 2, 3 or 4 types of maltreatment), this figure shows the proportion of participants who had a mental health condition at different levels of physical multimorbidity. Across all levels of physical multimorbidity, experience of maltreatment was associated with a higher prevalence of having any mental health condition.

Experiencing a greater number of types of childhood maltreatment was also associated with a higher prevalence of mental health conditions. This relationship was consistent across all levels of physical multimorbidity (Figure 4). This association remained clear after adjusting for the number of physical health conditions, as well as age, sex, socio-economic deprivation, BMI, smoking and alcohol (OR: 2.99; 99% CI: 2.54–3.51 for four types of maltreatment compared to none; full analysis is given in the Online Supplementary Material).

### Outcomes

Results of logistic regression analyses, restricted to participants with two or more LTCs, showing the adjusted relationship between experience of childhood maltreatment and outcomes at baseline assessment are shown in Tables 5 and 6. All results were adjusted for age, sex, socio-economic deprivation, BMI, smoking and alcohol frequency. Experience of each individual type of maltreatment as well as cumulative total of types of maltreatment were significantly associated with poorer self-rated health, loneliness, social isolation, widespread pain lasting >3 months and frailty (with the exception of the relationship between sexual abuse and social isolation, for which the 99% CI included the null). For each maltreatment category, increased frequency of maltreatment was associated with greater odds of reporting each outcome. When compared with no experience of maltreatment, participants experiencing all four types of maltreatment were more likely to be socially isolated (OR: 1.45; 99% CI: 1.03–2.05), and more than three times as likely to report poor self-rated health (OR: 3.56; 99% CI: 2.58–4.90), loneliness (OR: 3.16; 99% CI: 2.17–4.60), frailty (OR: 3.21; 99% CI: 2.04–5.05) and chronic widespread pain (OR: 3.19; 99% CI: 1.87–5.44). Results were similar when the same models were analysed for the full sample and additionally adjusted for number of LTCs. These are shown in the Online Supplementary Material.

**Table 5.** Maltreatment and outcomes complicating multimorbidity: Cross-sectional association between each type of maltreatment and outcomes.^a^

Maltreatment category	Outcome					
	Odds ratio (99% confidence interval)					
	Self-rated Health (poor)	Loneliness	Social isolation	Widespread pain for >3 months	Frailty phenotype	
					Pre-frailty	Frailty
Physical (yes/no)	1.48 (1.32–1.66)	1.56 (1.37–1.77)	1.17 (1.06–1.3)	1.51 (1.23–1.86)	1.18 (1.1–1.25)	1.54 (1.32–1.79)
Emotional (Yes/no)	1.8 (1.6–2.02)	2.01 (1.76–2.28)	1.35 (1.22–1.5)	1.7 (1.38–2.1)	1.31 (1.22–1.4)	1.7 (1.46–1.99)
Sexual (yes/no)	1.52 (1.31–1.76)	1.39 (1.17–1.65)	1.1 (0.96–1.26)	1.65 (1.28–2.11)	1.11 (1.02–1.21)	1.5 (1.24–1.81)
Neglect (yes/no)	1.83 (1.56–2.14)	1.87 (1.57–2.22)	1.32 (1.14–1.53)	1.76 (1.34–2.32)	1.28 (1.16–1.42)	2.06 (1.69–2.52)

^a^ All results adjusted for age, sex, socio-economic status, smoking and alcohol frequency. 99% confidence intervals are based on a Bonferroni correction to accommodate for multiple testing of five separate outcomes.

**Table 6.** Maltreatment and outcomes complicating multimorbidity: Cross-sectional association between number of types of maltreatment and outcomes.^a^

Maltreatment category	Outcome					
	Odds ratio (99% confidence interval)					
	Self-rated Health (poor)	Loneliness	Social isolation	Widespread pain for >3 months	Frailty phenotype	
					Pre-frailty	Frailty
0 (ref)	1	1	1	1	1	1
1	1.34 (1.18–1.52)	1.38 (1.19–1.59)	1.17 (1.05–1.29)	1.57 (1.26–1.97)	1.11 (1.04–1.18)	1.55 (1.32–1.82)
2	1.77 (1.52–2.07)	1.88 (1.58–2.23)	1.34 (1.17–1.53)	1.75 (1.32–2.33)	1.36 (1.24–1.48)	1.86 (1.52–2.29)
3	2.23 (1.8–2.76)	2.6 (2.06–3.28)	1.44 (1.17–1.76)	2.24 (1.54–3.27)	1.34 (1.16–1.55)	2.24 (1.68–2.98)
4	3.56 (2.58–4.9)	3.16 (2.17–4.6)	1.45 (1.03–2.05)	3.19 (1.87–5.44)	1.61 (1.22–2.12)	3.21 (2.04–5.05)

^a^ All results adjusted for age, sex, socio-economic status, smoking and alcohol frequency. 99% confidence intervals are based on a Bonferroni correction to accommodate for multiple testing of five separate outcomes.

## Discussion

This analysis demonstrates that child maltreatment is common and strongly associated not only with increased prevalence of multimorbidity but also total number of LTCs and complexity of multimorbidity. Thirty-three percent of participants reported at least one form of maltreatment. Each type of maltreatment, frequency of maltreatment and the total number of types of maltreatment experienced were each associated with a higher LTC count. Number of types of maltreatment experienced was also associated with increased prevalence of having any mental health condition. Furthermore, after controlling for LTC count, experience of maltreatment was associated with lower self-rated health, loneliness, social isolation, widespread chronic pain and frailty. Our findings therefore have important population- and individual-level implications. The prevalence of maltreatment may be an important factor contributing to multimorbidity. The consequences for individuals affected by child maltreatment are wide-ranging; our results suggest this includes increasing the complexity of multimorbidity and negatively impacting self-rated health.

Our findings are in keeping with the growing body of research looking at the impact of childhood adversity on future health and social outcomes.^2,3,37^ The first ACEs study^37^ was fundamental in establishing both the multiple domains affected (including many LTCs) and the impact of cumulative trauma. By taking a life course approach,^5^ we can recognise that exposure to factors in childhood, particularly at developmentally sensitive times, can be risk factors for the future development of disease. Our work, in conjunction with other studies,^2,3,7,9,10^ suggests that childhood maltreatment can have latent consequences, including the development of multimorbidity in adulthood. There are plausible biological pathways linking trauma (including child maltreatment) in childhood to increased allostatic load^7–9,38^ and changes in gene expression.^10^ In addition, experience of childhood adversity (including child maltreatment) increases the risk of unhealthy behaviours.^3^ These same biological pathways, and behavioural risk factors, that increase the development of different LTCs are likely to be implicated in the increased association with multimorbidity. Other studies have shown a link between childhood adversity and multimorbidity either as a binary variable (i.e. the presence or absence of two or more LTCs)^16,17^ or separating it into clusters based on combinations of chronic pain, mental health problems and physical health problems.^15^ When separating multimorbidity into clusters, physical abuse, sexual abuse or exposure to domestic violence as a child have been shown to increase the likelihood of painful and mental health conditions.^15^ Our findings expand on these associations, in particular showing an association between maltreatment and LTC count. We know that experience of multimorbidity, for patient and practitioner, is influenced by more than LTC count. Wider psychological and social factors have a significant impact not just on the work a patient must carry out but also their capacity to do so. Our findings suggest people experiencing childhood maltreatment are not only at risk of higher numbers of LTC in adulthood but also experiencing factors that will complicate self-management and practitioner work – with implications for the resources needed to manage these patients well. Our work also demonstrates an association between multimorbidity and psychosocial factors that make the patient experience more complex.

Multimorbidity is more prevalent and starts at an earlier age in areas of high socio-economic deprivation.^12^ This association is only partially explained by differences in risk factors such as smoking, obesity, diet, exercise and alcohol.^13^ Psychological comorbidity is also more common in the context of socio-economic deprivation,^12^ as is experience of child maltreatment and adversity.^39,40^ Given the multiple associations, experience of maltreatment (and other adversity in childhood) may be an important underlying factor in the social gradient of both multimorbidity and psychological comorbidity. Indeed, it is recognised that, due to the strong association between socio-economic position and childhood adversity, policies to tackle childhood adversity must also tackle the social determinants of health to be effective.^40^

Our findings of a strong association between maltreatment and complex multimorbidity have implications for how resources are allocated and health services designed. Patients experiencing multimorbidity with poor self-rated health, chronic pain, loneliness, social isolation, frailty or mental health conditions have more complex care needs and utilise more social and healthcare resources.

Our results also support the growing recognition of the potential benefit of trauma-informed services.^41,42^ Currently, these tend to be part of specialist teams or for specific populations (e.g. those experiencing considerable social exclusion).^43^ However, our findings show a high prevalence and considerable impact of maltreatment in a relatively affluent and healthy cohort (compared to the general population),^44^ suggesting the impact of child maltreatment in the wider population is potentially much greater as our prevalence figures are likely to be conservative. The work on trauma-informed services suggests that they are of benefit for people who have experienced trauma;^41,43^ there may be value in incorporating these trauma informed principles within more generalist healthcare environments (those most likely to be utilised by people with complex multimorbidity).^41,42^ In addition, training of general healthcare staff around the long-term physical and emotional impact of trauma may aid care for this patient group if principles are applied to whole populations, even if maltreatment is not disclosed. Resources and training are required to enable health professionals and others to support the complex needs of this population. This is the rationale behind the transforming psychological trauma national framework being rolled out by NHS Scotland.^42^ We would suggest there is a need to not only increase the amount of specialist services offered and to consider whether trauma-informed principles and training should be applied to more mainstream services.

As well as ensuring adequate support for patients experiencing complex multimorbidity, the importance of prevention is paramount. Investing in prevention and support of early childhood adversity could result in improved health outcomes in the future. Our results add to the evidence that efforts to mitigate the impact of childhood adversity should be seen as public health measures.^2^ The growing evidence of an association between child maltreatment and multimorbidity, particularly complex multimorbidity as indicated here, highlights the need to provide ongoing support for adults who have experienced childhood adversity to help break the cycle of intergenerational adversity.^2^

Strengths of this study include the large sample size which allowed a more detailed analysis of numbers of LTCs and frequency of maltreatment than in previous studies,^16,17^ consideration of both frequency and type of maltreatment, and adjustment for a range of potential confounding variables. Assessing the number of LTCs as well as factors complicating multimorbidity allowed a more nuanced assessment of the relationship between maltreatment and multimorbidity than in previous studies. However, UK Biobank is not a representative population sample. Participants are, on average, more affluent and have a lower prevalence of multimorbidity than the population in general. Furthermore, the assessment of experience of maltreatment was part of a follow-up questionnaire completed by a subset of the original baseline cohort. This introduces bias by excluding people who had died in the intervening period or did not respond. In addition, the follow-up questionnaire, while based on a validated questionnaire,^34^ only asked one question for each category of maltreatment. Our findings cannot be used to infer the prevalence of maltreatment in the wider population and are likely to be conservative. Furthermore, the potential exposure was enquired about 10 years on from the other variables (including our outcome, multimorbidity), introducing potential responder and recall bias. Specifically, given that people who responded to the follow-up questionnaire were on average healthier and less socio-economically deprived than the cohort in general, people responding may be less likely to have experienced maltreatment. It is also possible that unrecorded factors occurring between baseline and follow-up could have some influence on how people responded to some questions. A further limitation was that other adverse experiences in childhood (parental incarceration or abandonment, domestic violence, parental mental illness or addiction) were not included in the questionnaire. This limits comparability with other literature that has used the standardised questionnaires, and our findings can only be considered in the context of child maltreatment rather than wider childhood adversity. However, despite this, the observed relationships between exposures and outcomes are clear and likely to be transferable. LTCs were assessed by self-report; however, respondents were supported by a study nurse in providing these data. There may be inaccuracies in self-report of some conditions (e.g. chronic kidney disease); however, self-report may be superior for other conditions (e.g. chronic back pain). Importantly, both inclusion of and accuracy of recording conditions such as dementia may be limited by self-report. Cognitive impairment may also impact the reliability of the follow-up questionnaire from which maltreatment was assessed. We used an area-based marker of socio-economic deprivation; however, this may overlook important individual indicators of socio-economic deprivation resulting in residual confounding.

Our findings demonstrate association only, not causation. However, they are consistent with the growing body of scientific literature linking exposure to toxic stress in childhood to multiple health consequences in later life. The assessment of experience of maltreatment was not concurrent with the assessment of multimorbidity; however, given that the questions specifically addressed maltreatment in childhood, there is not a question over the temporal relationship between exposure (i.e. maltreatment) and outcome (i.e. multimorbidity).

## Conclusion

Experience of child maltreatment affects a high proportion of people, with a minority experiencing frequent and multiple maltreatment. Multiple and frequent maltreatment is strongly linked to both a higher LTC count in adulthood and a range of factors which complicate management. A multifaceted response, combining population and individual-level interventions, is required if child maltreatment is to be reduced and its impact mitigated. As health services look to respond to increasing multimorbidity, better understanding of potentially preventable precursors such as maltreatment, and its impacts, is required across a range of services – both specialist and generalist. Greater recognition and recording of maltreatment, alongside sensitive and appropriate identification of people affected by maltreatment, with personalised response, is required, particularly in healthcare settings dealing with high levels of multimorbidity.

## Supplemental material

Supplemental Material, MM_and_child_maltreatment_supplementary_material - Association between childhood maltreatment and the prevalence and complexity of multimorbidity: A cross-sectional analysis of 157,357 UK Biobank participants Click here for additional data file. Supplemental Material, MM_and_child_maltreatment_supplementary_material for Association between childhood maltreatment and the prevalence and complexity of multimorbidity: A cross-sectional analysis of 157,357 UK Biobank participants by Peter Hanlon, Marianne McCallum, Bhautesh Dinesh Jani, Ross McQueenie, Duncan Lee and Frances S Mair in Journal of Comorbidity
