# Supplementary material for: Association between childhood maltreatment and the prevalence and complexity of multimorbidity: A cross-sectional analysis of 157,357 UK Biobank participants
Source: J Comorb. 2020 Jul 31;10:2235042X10944344. doi: 10.1177/2235042X10944344 (PMC7416137; doi:10.1177/2235042X10944344)
Supplement: Supplemental Material, MM_and_child_maltreatment_supplementary_material - Association between childhood maltreatment and the prevalence and complexity of multimorbidity: A cross-sectional analysis of 157,357 UK Biobank participants [file MM_and_child_maltreatment_supplementary_material.pdf]

Association between childhood maltreatment and the prevalence and complexity of multimorbidity: a cross sectional analysis of 157,357 UK Biobank participants

Supplementary Appendix

Peter Hanlon, Marianne McCallum, Bhautesh Dinesh Jani, Ross McQueenie, Duncan Lee, Professor Frances S Mair

Baseline characteristic comparing participants who completed the mental health follow-up questionnaire (and were therefore included in the analysis) and those who did not.

Missing versus non-missing participants		
Variable	Completed mental health follow-up questionnaire	Did not complete mental health follow-up questionnaire
**Age**<br>Median (IQR)	57 (50-62)	58 (50-64)
**Sex**		
Female (%)	89097 (57)	184369 (53)
**Socioeconomic status (%)**		
Quintile 1	35683 (23)	65004 (19)
Quintile 2	33661 (21)	66458 (19)
Quintile 3	32510 (21)	67902 (20)
Quintile 4	30773 (20)	69622 (20)
Quintile 5	24531 (16)	75869 (22)
Missing	199	428
**Smoking (%)**		
Current	11339 (7)	41650 (12)
Previous	55281 (35)	117817 (34)
Never	90360 (58)	183241 (54)
Missing	377	2575
**Alcohol (%)**		
Never/occasional	23355 (15)	75335 (22)
1-3/month	17265 (11)	38608 (11)
1-4/week	80039 (51)	164745 (48)
Daily/almost daily	36573 (23)	65217 (19)
Missing	125	1378
**BMI**		
<18.5	896 (0.6)	1730 (0.5)
18.5-25	58299 (37)	99168 (29)
25-30	65820 (42)	148447 (44)
>30	31329 (20)	91131 (27)
Missing	1013	4807
**No. of LTCs**		
0 LTC	61173 (39)	111428 (32)
1 LTC	52741 (34)	110971 (32)
2 LTCs	27033 (17)	68198 (20)
3 LTCs	10663 (7)	32460 (9)
4 or more LTCs	5425 (4)	20703 (6)
Missing	322	1523

Adjusted relationship between number of types of maltreatment and presence of mental health comorbidity

Logistic regression assessing association between childhood maltreatment (number of types of experiences) and presence of any mental health comorbidity (adjusted for age, sex, socioeconomic status, BMI smoking, alcohol intake, and number of physical LTCs)	
Number of types of abuse	OR (95% CI)
0	(ref)
1	1.34 (1.27-1.40)
2	1.85 (1.74-1.97)
3	2.14 (1.94-2.35)
4	2.98 (2.54-3.51)

Relationship between maltreatment and outcomes in whole sample, adjusted for number of long-term conditions as well as age, sex, socioeconomic deprivation, BMI, smoking and alcohol intake.

**Cross sectional association between each type of maltreatment and outcomes**						
**Maltreatment category**	**Outcome<br>Odds ratio (99% confidence interval)**					
	**Self-rated Health (poor)**	**Loneliness**	**Social isolation**	**Widespread pain for >3 months**	**Frailty phenotype**	
					**Pre-frailty**	**Frailty**
Physical (yes/no)	1.36 (1.23-1.51)	1.53(1.41-1.65)	1.13 (1.07-1.20)	1.37 (1.15-1.63)	1.15 (1.11-1.19)	1.49 (1.32-1.69)
Emotional (Yes/no)	1.66 (1.50-1.84)	1.95 (1.80-2.11)	1.31 (1.23-1.40)	1.55 (1.30-1.85)	1.24 (1.20-1.29)	1.68 (1.48-1.91)
Sexual (yes/no)	1.32 (1.16-1.51)	1.36 (1.21-1.51)	1.10 (1.01-1.19)	1.51 (1.22-1.87)	1.15 (1.09-1.21)	1.33 (1.13-1.56)
Neglect (yes/no)	1.75 (1.52-2.01)	1.98 (1.76-2.21)	1.23 (1.12-1.35)	1.59 (1.25-2.01)	1.26 (1.19-1.34)	1.99 (1.68-2.37)
**Cross sectional association between number of types of maltreatment and outcomes**						
**Maltreatment category**	**Outcome<br>Odds ratio (99% confidence interval)**					
	**Self-rated Health (poor)**	**Loneliness**	**Social isolation**	**Widespread pain for >3 months**	**Frailty phenotype**	
					**Pre-frailty**	**Frailty**
0 (ref)	1	1	1	1	1	1
1	1.24 (1.11-1.39)	1.41 (1.29-1.53)	1.12 (1.05-1.18)	1.43 (1.19-1.72)	1.12 (1.08-1.16)	1.43 (1.26-1.64)
2	1.65 (1.44-1.89)	1.88 (1.69-2.09)	1.24 (1.14-1.34)	1.59 (1.25-2.02)	1.29 (1.22-1.36)	1.85 (1.56-2.19)
3	2.03 (1.67-2.46)	2.58 (2.20-3.00)	1.41 (1.24-1.61)	2.12 (1.55-2.92)	1.37 (1.25-1.49)	2.12 (1.66-2.72)
4	2.70 (1.98-3.69)	3.56 (2.75-4.61)	1.60 (1.27-2.03)	2.14 (1.28-3.57)	1.53 (1.28-1.83)	2.65 (1.76-4.00)
All results adjusted for age, sex, socioeconomic status, smoking, alcohol frequency, and multimorbidity count.<br>99% confidence intervals are based on a Bonferroni correction to accommodate for multiple testing of five separate outcomes.						
